# The Finger Nails in Disease

**Published:** 1886-06

**Authors:** 


### The Finger Nails in Disease.

The subject of the finger-nails in disease is a very interesting one and will prove of service to you in many instances. In all morbid actions that interfere with nutrition, the finger-nails share, and the marks of such irregularities are left for some time on the nails. A mere pustule on the finger will more or less check the growth of the nail, and if this pustule be severe enough to cause a febrile condition, all the nails will be affected. If a man breaks his arm, the swelling of the parts will so interfere with nutrition as to affect the nails, and we can detect whether one has been really sick or deceiving us, for these marks will last as long as nine months, or until the whole nail has grown out. Of course there are no specific indications of particular diseases, but the indications of some trouble that has affected nutrition will be found. If the patient has had typhoid fever, pneumonia, pleurisy, or acute rheumatism, the evidence will be found in the nail.

Again, a man may work in a dye-shop, or some similar place that will stain his nails; if he tells you that he has been sick and unable to work for some time, if the whole of his nails are stained, you will know that he has been deceiving you, for if he has been away from work for some time, that portion of his nails which has since grown will have the natural color, while the extremities will be stained.

You will often come in contact with persons from whom you can get no history; maybe they have been found unconscious, and you can tell whether they have been sick for some time, or whether the attack is sudden, from the nails.

Washing the hands and abrasion from ordinary use, will separate the skin from the base of the nail, while if for some time the hands have not been washed or used this will be unbroken. Here we have one who is convalescent from typhoid fever; during the disease his nails did not grow; now they are growing, and we know that he is convalescent.

The change referred to consists in a depression, extending one-third of the way through the nail, commencing at the matrix and advancing as the nail grows, until it is finally discharged from the tip, when it reaches which, the end of the nail is liable to be very brittle and break. This phenomenon is on the same principle as with the hair, which, when it commences to grow from its socket, in convalescence, is brittle from innutrition, and breaks. In such diseases as rheumatism, where there is a sudden onset, a sudden rise in temperature, which is maintained, this effect is more marked, giving us a very abrupt depression, while in diseases of slower inception, as typhoid fever, it is more gradual, the nail gradually sloping on either side toward the depression. When the nail is growing very slowly during the disease the skin about the matrix adheres to it and is dragged forward, but when convalescence is established, and a more rapid growth commences, the nail shoots forward and tears away from the skin. If, as is sometimes the case, you can not see this depression, you can feel it. Sometimes there is no depression, but a more or less marked white line extends across the nail. If we have two attacks of fever, the one following rapidly on the other, as in relapse from typhoid, there will be two lines, which may be pinkish near the root, but becomes white as they grow out.

During an epidemic of relapsing fever in this house, in 1869 and 1870, I had the disease myself, and I first noticed these white lines in my own person and showed them to my medical friends, none of whom could offer an explanation.

I soon noticed the same peculiarity in typhoid, and looking the matter up, I found that some old French physicians had recorded cases of malignant fever in which the nails broke off, and in some cases came off entire. I think we do not have such types of disease to-day, for we certainly do not have the nails falling off.

Vogel also speaks of this peculiarity of the nails, but he falls into the error of supposing it an elevation instead of a depression.

I have taken off the end of the finger in patients who have died, and made sections, when I was enabled to see a marked checking of the growth of the nail cells.

These observations are important from a medico-legal point of view, and it is well to remember that the marks will last the longest on the nail of the great toe.—*Medical and Surgical Reporter.*

